# EGFR signaling confers resistance to BET inhibition in hepatocellular carcinoma through stabilizing oncogenic MYC

**DOI:** 10.1186/s13046-019-1082-6

**Published:** 2019-02-15

**Authors:** Yalei Yin, Mingju Sun, Xi Zhan, Changqing Wu, Pengyu Geng, Xiaoyan Sun, Yunsong Wu, Shuijun Zhang, Jianhua Qin, Zhengping Zhuang, Yang Liu

**Affiliations:** 1 CAS Key Laboratory of Separation Science for Analytical Chemistry, Scientific Research Center for Translational Medicine, Dalian Institute of Chemical Physics, Chinese Academy of Sciences, Dalian, 116023 China; 2 School of Life Science, Dalian University, Dalian, 116023 China; 3 Surgical Neurology Branch, National Institute of Neurological Disorders and Stroke, National Institutes of Health, Bethesda, MD 20892 USA; 4 College of Chemistry and Molecular Sciences, Wuhan University, Wuhan, 430072 China; 5 Department of Hepatobiliary and Pancreatic Surgery, The First Affiliated Hospital of Zhengzhou University, Zhengzhou, Henan China; 6 Open and Key Laboratory of Hepatobiliary and Pancreatic Surgery and Digestive Organ Transplantation at Henan Universities, The First Affiliated Hospital of Zhengzhou University, Zhengzhou, Henan China; 7 Zhengzhou Key Laboratory of Hepatobiliary and Pancreatic Diseases and Organ Transplantation, Zhengzhou, Henan China; 8 Henan Key Laboratory of Digestive Organ Transplantation, Zhengzhou, Henan China; 9 University of Chinese Academy of Sciences, Beijing, 100049 China

**Keywords:** Bromodomain, JQ1, MAPK pathway, MYC, EGFR mutation, Sorafenib

## Abstract

**Background:**

The bromodomain and extra-terminal domain (BET) inhibitor is a type of anti-tumor agent, currently being evaluated in phase I and II clinical trials for cancer therapy. It can decrease MYC expression levels and cause effective anti-tumor effects in diverse human cancers. However, its cytotoxic effect and related mechanisms of drug resistance are poorly understood in hepatocellular carcinomas (HCC). Here, we investigated the anti-tumor effects of BET inhibitor on HCC and the molecular mechanisms involved in its associated drug resistance.

**Methods:**

We assessed the cytotoxicity of BET inhibitor on HCC cells compared with sorafenib by cell viability assay, metastasis assay and reproduced the anti-tumor effect in xenograft mouse model. In addition, the molecular mechanisms involved in drug resistance on JQ1-resistant HCC cells were revealed by western blotting, qRT-PCR, whole exome-sequencing and gene-editing technology. Finally, with specific inhibition of EGFR or ERK activity by interference RNAs or inhibitors, the efficacy of the synergistic treatment was investigated using cell viability assay, colony formation, apoptosis and xenograft mouse model.

**Results:**

We found that JQ1, a commonly used BET bromo-domain inhibitor, offered a better anti-tumor response than sorafenib in MYC-positive HCC cells by inducing apoptosis in vitro and in vivo. Unlike sorafenib, JQ1 treatment significantly impaired mitochondrial respiration and glycolysis in HCC cells. Importantly, we revealed that MAPK activation by a previously undescribed activating mutation of EGFR-I645L, was critical for JQ1 sensitivity through stabilizing oncogenic MYC protein in JQ1-resistant HCC cells. Inhibition of either EGFR or ERK activity overcame the JQ1 resistance and significantly decreased MYC protein level in vitro and in vivo.

**Conclusion:**

Since MYC amplification is frequently identified in HCC, co-occurring with EGFR amplification, our findings suggest that targeting EGFR signaling might be essential for JQ1 therapy in advanced HCC.

**Electronic supplementary material:**

The online version of this article (10.1186/s13046-019-1082-6) contains supplementary material, which is available to authorized users.

## Background

Sorafenib, a multiple kinase inhibitor, is currently the standard first-line drug approved by the Food and Drug Administration (FDA) for advanced hepatocellular carcinoma (HCC) [1–3]. Although sorafenib improves overall survival of HCC patients, the tumor response is generally poor [4–7]. Recently, lenvatinib and regorafinib, were approved as the first-line and second -line treatment for HCC, respectively [8]. However, similarly to sorafenib, both of these two drugs inhibit tumor angiogenesis by interfering with multiple receptor tyrosine kinases (VEGFR, PDGFR), potentially leading to more severe intratumor hypoxia and drug resistance [9–11]. Nivolumab, a checkpoint-inhibitor was approved as second-line drug for the treatment of advanced HCC patients, the safety and efficacy are still being evaluated in clinic [12, 13]. Therefore, there is an urgent need to develop novel therapeutic drugs for HCC treatment.

Previous studies showed that high expression levels of MYC, a critical regulator of cell proliferation and metabolism [14–16], were observed in about 20% of HCC tumors and significantly correlated with tumorigenesis [17, 18]. Therefore, inhibition of MYC expression by small molecules has been shown to have great therapeutic potential for HCC treatment [19]. Bromodomain-containing protein 4 (BRD4) is a member of the bromodomain and extra-terminal (BET) family proteins, recruiting transcriptional regulatory proteins for binding to transcription start sites of genes, such as MYC, BCL2 and BCL6 genes [20, 21]. JQ1, a specific and competitive inhibitor of BRD4, can decrease MYC expression levels and induce apoptosis in diverse human cancers, including medulloblastoma, lung adenocarcinoma, glioblastoma, and myeloid leukemia [22–24]. Although JQ1 is still being evaluated in phase I and II clinical trials for the treatment of advanced malignancies [25], emerging evidence demonstrated that the anti-tumor effect of JQ1 was limited in tumor cells with high level of MYC protein [26, 27]. It has been documented that PI3K/AKT/mTOR and MAPK/ERK activation may be involved in intrinsic resistance of JQ1 in tumor cells [28, 29]. Furthermore, recent study showed that BRD4 could be considered as an attractive therapeutic target for HCC treatment [30]. However, since the sensitivity of this drug varies across a number of differential HCC cell lines, it is crucial to understand the molecular basis of JQ1 resistance in HCC cells with high level of MYC expression.

In this study, we sought to determine the anti-tumor effects of the BET inhibitor and the molecular mechanisms adopted by HCC cells against this drug. As a result, we demonstrated that the anti-tumor effects of JQ1 were more potent than those of sorafenib in MYC-positive HCC cell lines. In addition, whole exome-sequencing (WES) revealed a novel activating EGFR mutation-I645L, mediating JQ1 resistance by activating MAPK pathway and stabilizing MYC protein in HCC cells. Combined treatment of JQ1 with EGFR or ERK inhibitor significantly resulted in tumor growth inhibition in vitro and in vivo*.* Our findings suggest that combination of JQ1 with EGFR/MAPK inhibition may be an attractive therapeutic strategy in advanced HCC with EGFR activation.

## Materials and methods

### Cell lines, plasmid transfection, viral infection

The HCC cell lines Hep3B, HCCLM3(LM3), HuH7, HB611, HepG2, SMMC7721, MHCC97-L (97-L), MHCC97-H (97-H), PLC/PRF/5 and BEL-7402 were purchased from the ATCC and maintained in Dulbecco’s modified Eagle’s medium or RPMI-1640 medium supplemented with 10% fetal bovine serum at 37 °C in a humidified atmosphere with 5% CO_2_. The EGFR-WT and EGFR-I645L cDNAs were obtained from 97-L and 97-H cells following RNA isolation and subsequent reverse transcription PCR (Takara, Japan). cDNAs of wild type and mutanted EGFR were cloned into pCDH-EF1-coGFP-puro lentiviral vector (CD513B1, SBI Inc., Mountain View, CA, USA) using XbaI and NheI restriction sites, respectively. Lentivirus based shRNA against EGFR was purchased from Origene Inc. (TL320326, Rockville, MD, USA). For plasmid transfection, Lipofectamine 2000 was used according to the standard protocol. Plasmids were co-transfected with the packaging plasmid (TR30022, Origene, Rockville, MD, USA) into 293 T cells to generate the viral supernatant, then the viral supernatant infected cells. Stable HCC cell lines were established in culturing with 4 μg/ml puromycin (Thermo-Fisher, MA, USA).

### Reagents

JQ1, Sorafenib (Sora), AZD6244 (AZD), SCH772984 (SCH) and Erlotinib (ERL) were purchased from Selleck (Houston, TX, USA) and diluted in DMSO. Cycloheximide (CHX) was purchased from MCE (Shanghai, China). Antibodies against BRD4 (#13440), MYC (#5605), p-MYC-Ser62 (#13748), PARP (#9542), cleaved-PARP (#5625), cleaved-Caspase3 (#9664), ERK1/2 (#4695), p-ERK1/2(T202/Y204) (#4370), AKT (#2920), p-AKT(S473) (#4060), EGFR (#4267), p-EGFR(Tyr1068) (#3777), cleaved-Caspase3 (#9664) and GAPDH (#5174) were purchased from Cell signaling Technology (CST, Boston, USA). Antibodies against p-MYC-Thr58 (#28842), Ras (#52939) and p-MYC-Ser62 (#185656) for immunohistochemistry were purchased from abcam (Cambridge, UK). The β-Actin antibody was purchased from Santa Cruz Inc.(Dallas, TX, USA).

### Cell viability assay

For the cell viability assay, 5.0 × 10^3^ cells per well were plated in 96-well plates and cultured in a 5% CO_2_ incubator. After 24 h, compounds were added in serial dilutions. 48 h later, cell viability was assessed using the Cell Titer-Glo® Luminescent Cell Viability Assay (Promega, Madison, WI, USA) following the protocols provided by the manufacturers.

### Extracellular acidification rate (ECAR)

ECAR assay was determined by Seahorse XF96e analyzer (Seahorse Bioscience, USA) according to the manufacturer’s instructions. Briefly, 1.0 × 10^4^ cells were plated per well in a 24-well XF cell culture microplate. ECAR was measured in XF base medium containing 4 mM glutamine (pH 7.35) following sequential additions of glucose (10 mM), oligomycin (1 mM) and 2-DG (50 mM). Data were analyzed by a Seahorse XF Glycolysis Stress Test Report Generator.

### Colony formation assay

For colony formation assay, 1.0 × 10^4^ cells were plated into 6-well dishes in 2 ml of medium containing 0.3% agarose, overlaid with 2 ml of 0.5% agarose. DMEM was added on top, and maintained in a humidified atmosphere with 5% CO_2_ at 37 °C for 6 weeks. Then colonies were stained with crystal violet, and the number of colonies was counted.

### Quantitative real-time PCR

Total RNA was isolated from cell pellets with the Trizol® Reagent (Takara, Japan) and reverse-transcribed into cDNA using SYBR premix EX Taq (Takara, Japan) and specific primers. Gene expression level was assessed by qRT-PCR using the CFX96 Touch™ Real-Time PCR Detection System according to the manufacturer’s instructions. GAPDH was used as the loading control. MYC primer sequences were: F:5’-CGTCCTCGGATTCTCTGCTCTC-3′, R: 5’-GCGCTGCGTAGTTGTGCTGAT-3′. GAPDH primer sequences were: F: 5’-CTTCGCTCTCTGCTCCTCCTGTTCG-3′, R: 5’-ACCAGGCGCCCAATACGACCAAAT-3′.

### Annexin V/ Propidium iodide (PI) double-staining assay

Annexin V-FITC apoptosis detection kit (BD biosciences, USA) was used to assess the apoptosis. Annexin V and PI staining was performed according to the manufacturer’s protocol. Briefly, 1.0 × 10^5^ cells in 200 μL 1× Binding Buffer were incubated with 10 μL FITC Annexin V and 5 μL PI for 30 min at room temperature. Stained cells were detected using a FACScan flow cytometer (BD biosciences) and data was collected using FlowJo software.

### Western blotting

Immunoblotting was carried out using standard protocol. Briefly, cells were lysed in ice-cold 1× RIPA lysis buffer, and protein concentrations were determined by bicinchoninic acid (BCA, Tiangen, China). Cell lysates were loaded on precast 4 to 10% NuPAGE Novex 4 to 12% Bis-Tris Protein Gels (Life technologies, Carlsbad, CA). Then the proteins were transferred onto PVDF membranes, subsequently blocked and incubated with primary antibodies overnight at 4 °C. After washing, the membranes were probed with HRP conjugated rabbit-anti-goat secondary antibodies (1:4000) for 2 h at room temperature. Targeted proteins were visualized using enhanced chemiluminescence (Thermo-Fisher, MA, USA) on Hyperfilm (GE Healthcare, MA, USA).

### Xenograft mice model

All animal procedures were performed in accordance with the NIH Guide on the Care and Use of Laboratory Animals and approved by the Institutional Animal Care and Use Committee of the Dalian Medical University. 5 × 10^6^ Cells suspended in a mixture of Matrigel and 1× PBS were implanted subcutaneously into the flanks of 5-week-old CB17/SCID mice. For the experiment to determine JQ1 and sorafenib in vivo cytotoxicty, we injected BEL-7402 and 97-L cells into immunodeficient nude mice to generate HCC xenograft tumors, respectively. When tumors nodules had been formed and reached a volume of 100 mm^3^, the xenograft mice were divided into three groups and treated with vehicle, JQ1 or sorafenib respectively at 50 mg/kg every 2 days. For the experiment to determine the anti-tumor effect of combined treatment in vivo, 97-H cells were implanted into immunodeficient nude mice for subcutaneous xenograft tumor formation. When tumors nodules reached a volume of 100 mm^3^, mice were divided into four groups and treated with Vehicle, JQ1 (50 mg/kg), ERL (50 mg/kg) or a combination of both drugs every 2 days. Tumors were measured with calipers and tumor volume V was calculated using the equation: V = π/6 × length × width^2^.

### Immunohistochemistry

Tumor xenografts were embedded in Tissue-Tek OCT (Sakura, USA) for frozen liver sections (thickness 10 μm) with a standard procedure. For immunohistochemistry (IHC), the slides were fixed with 4% paraformaldehyde. 3% peroxidase and 20% fetal bovine serum were diluted in 1× PBS to block endogenous peroxide activity and nonspecific binding. The slides were incubated with primary antibodies overnight at 4 °C followed by biotin-streptavidin HRP detection systems (ZSGB-BIO, China). Hematoxylin dye was used as counter stain. The slides were examined with an Olympus BX61 microscope with cellSens Standard Software Version 1.6 (Olympus Corporation, Tokyo, Japan).

### Statistical analysis

Statistical analyses were performed using Microsoft Excel software. Results were representative of three independent experiments with three replicates. Student’s t-tests were used to analyze the statistical significance of differences between two groups. *P*-value of < 0.05 was considered statistically significant in all cases and indicated by one asterisk. Error bars shown in the figures represent s.d.

## Results

### BET inhibitor inhibits tumor growth more effectively than sorafenib in MYC-positive HCC cells

**Fig. 1:**
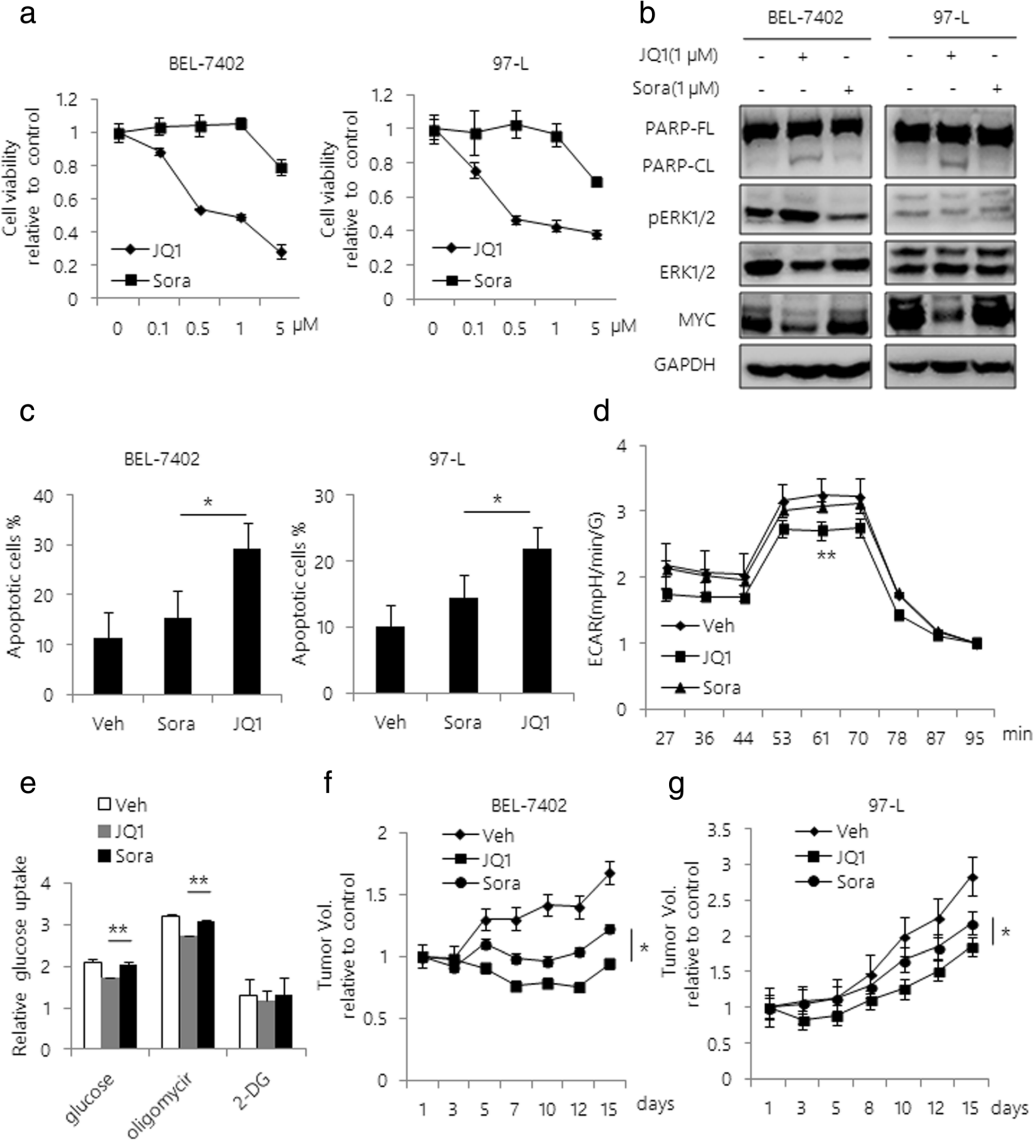
BET inhibitor inhibits tumor growth more potently than sorafenib in MYC- positive HCC cells. **a** Cell viability curves are shown for varying doses of JQ1 or sorafenib in BEL-7402 and 97-L cells. Cell viability was determined at 48 h after treatment using Cell Titer-Glo. Values were independently normalized to the untreated cells. **b** Western blot analysis of BEL-7402 and 97-L cell lysates after treatment with JQ1 or sorafenib for 48 h. The antibodies used are indicated. GAPDH was used as a loading control. **c** BEL-7402 and 97-L cells were treated with either JQ1 or sorafenib for 48 h. Apoptosis was assessed by Annexin V / PI double staining. Quantification of apoptotic cells was determined based on Annexin V positive cells. ECAR (**d**) and Glucose uptake values (**e**) was measured after JQ1 or sorafenib treatment for 24 h by the Seahorse XF Glycolysis Stress Test. BEL-7402 (**f**) and 97-L cells (**g**) (5 × 10^6^ each) were injected into the flanks of CB17/SCID mice. Mice were treated with vehicle, JQ1 or sorafenib at 50 mg/kg every 2 days. Tumor volumes were monitored during the treatments. Tumor volumes were assessed using caliper measurements (π/6 × length ×width^2^); *n* = 5 for each group. Data are presented as mean ± s.d. *p < 0.05, **p < 0.01

First, we investigated whether MYC amplification is correlated with clinical characteristics in different public transcriptomic datasets [31]. We found that high levels of MYC mRNA are associated with high risk for tumor pathogenesis and poor prognosis in HCC (Additional file 1: Figure S1a and b). Next, to evaluate the anti-tumor potential of BET inhibition on HCC cells, we investigated the cytotoxicity of JQ1 on HCC cells in comparison with sorafenib. The results showed a sharp reduction of cell viability in response to JQ1 in BEL-7402 and 97-L cell lines, while sorafenib treatment caused a mild growth inhibition (Fig. 1a). As expected, JQ1 treatment significantly decreased levels of MYC in both cell lines and induced cellular apoptosis supported by increase in PARP cleavage and Annexin V + population, while sorafenib treatment did not induce cellular apoptosis in these cells (Fig. 1b, c and Additional file 2: Figure S2). Since the increased dependency of anaerobic glycolysis is critical for HCC pathogenesis [32], we examined the effect of JQ1 and sorafenib on cellular glycolysis in HCC. We found that JQ1 significantly impaired mitochondrial respiration by attenuating glycolytic metabolism in HCC, supported by decreased Extracellular Cidification Rate (ECAR) comparing to sorafenib-treated cells (Fig. 1d and e). Next, to determine if JQ1 could reproduce the anti-tumor effect in HCC mice model, we injected BEL-7402 and 97-L cell lines subcutaneously into immunodeficient nude mice to generate HCC xenograft tumors. We found that JQ1 was able to reduce in vivo tumor growth more efficiently than sorafenib (Fig. 1f and g). The average tumor volumes were significantly smaller in JQ1 treatment group than sorafenib treated mice group (Additional file 3: Figure S3a). Furthermore, in consistent with our in vitro results, both decreased levels of MYC and induction of apoptosis were observed in JQ1 treated tumor xenografts by IHC staining (Additional file 3: Figure S3b and c). In contrast, we did not observe significant apoptosis in sorafenib treated mice group.

### JQ1 selectively inhibits HCC cell tumor growth in vitro

**Fig. 2:**
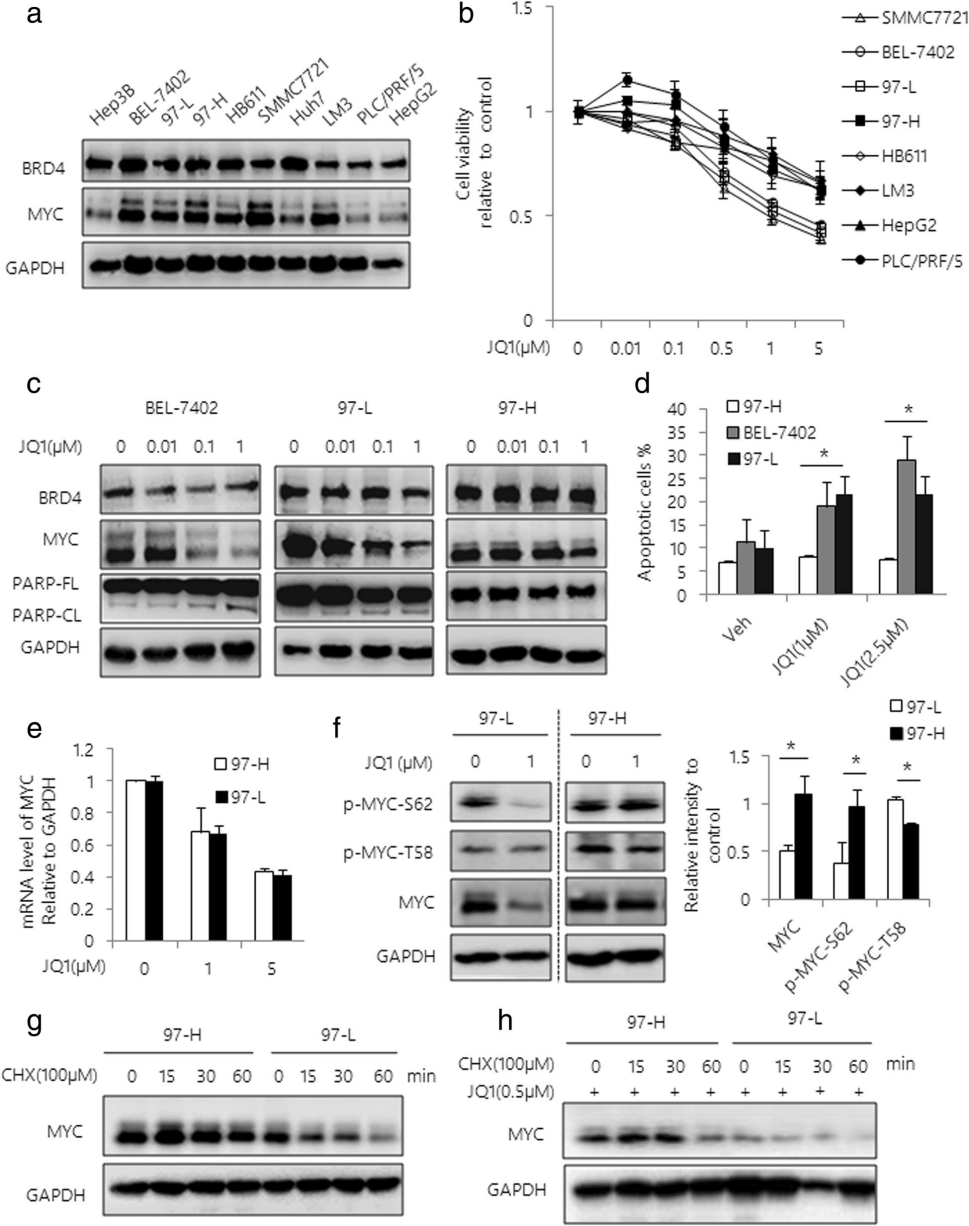
JQ1 selectively inhibits HCC cell tumor growth. **a** Western blot analysis of BRD4 and MYC expression in 10 HCC cell lines. GAPDH was used as a loading control. **b** Cell viability curves are shown for serial dilutions of JQ1 in 8 HCC cell lines. Cell viability was determined at 48 h after treatment using Cell Titer-Glo. **c** Western blot analysis of BEL-7402, 97-L and 97-H cells treated with serial dilutions of JQ1 for 48 h. Total lysates were subjected to the indicated antibodies. **d** BEL-7402, 97-L and 97-H cells were treated with varying dose of JQ1 for 48 h. Apoptosis was assessed by Annexin V / PI double staining. Quantification of apoptotic cells was determined based on Annexin V positive cells. **e** 97-L and 97-H cells were treated with JQ1 for 48 h. MYC mRNA expression level was analyzed by qRT-PCR. **f** Western blot analysis of 97-L and 97-H cells treated with 1 μM JQ1 for 48 h. Expressions of MYC, p-MYC-Ser62 and p-MYC-Thr58 were examined. Band intensities were quantified by Image J software and graphed at the right side. GAPDH was used as a loading control. Western blot analysis of 97-L and 97-H cells incubated with 100 μM CHX in the absence (**g**) or presence (**h**) of JQ1. Data are presented as mean ± s.d. *p < 0.05

To further investigate the anti-tumor effect of JQ1 in a variety of HCC cells, we assessed the expression levels of BRD4 and MYC in ten HCC cell lines. The relatively high levels of MYC and BRD4 expression were identified in 6 out of 10 cell lines, including BEL-7402, 97-L, 97-H, HB611, SMMC-7721 and HCCLM3 cells (Fig. 2a). Furthermore, eight HCC cell lines were tested for the cytotoxicity of JQ1. We found that the cytotoxic effect of JQ1 was positively correlated with MYC status (Fig. 2b). Notably, JQ1 significantly induced cytotoxicity in a dose dependent manner on HCC cells with relatively high MYC levels, including SMMC-7721, BEL-7402 and 97-L cells (Fig. 2b). The on-target effect of JQ1 was confirmed by western blot analysis, showing the dose-dependent reduction of MYC levels (Fig. 2c). In addition, induction of cellular apoptosis by JQ1 was observed by PARP cleavage, and further supported by Annexin V / PI double staining (Fig. 2d and Additional file 4: Figure S4). While we found that JQ1 failed in decreasing the cell activity on HCC cells with lower MYC level, such as HepG2 and PLC/PRF/5 cells. Interestingly, we observed differential cytotoxic response to JQ1 in 97-L and 97-H cells, which are originated from the same HCC tumors [33]. Unlike 97-L, 97-H cells were relatively insensitive to JQ1 and were characterized by sustained MYC expression regardless of JQ1 treatment. To determine the possible mechanisms involved in the cellular resistance to JQ1, we assessed the mRNA level of MYC in JQ1-treated cells by qRT-PCR, showing similar reduction of MYC mRNA levels in both of these two cell lines (Fig. 2e). These results led us to examine an involvement of posttranscriptional regulation of MYC protein in JQ1-treated 97-H cells. Indeed, we observed sustained phosphorylation of MYC at Ser62 site (S62), which is crucial for preventing oncogenic MYC from proteasomal degradation [34]. In contrast, the level of phosphorylation of MYC at Thr58 (T58), which is critical for degradation of MYC protein, was significantly decreased by JQ1 in 97-H cells. While there was no significant change of level of p-MYC-Thr58 in 97-L cells (Fig. 2f). To further assess the difference of MYC stability between these two cells, we treated the cells with cycloheximide (CHX), a molecule to block protein synthesis. As expected, we found that CHX significantly reduced MYC protein level in 97-L cells, but not in 97-H cells with or without treatment of JQ1, suggesting the enhanced protein half-life of MYC in 97-H cells (Fig. 2g and h).

### MAPK activation is critical for JQ1 sensitivity

**Fig. 3:**
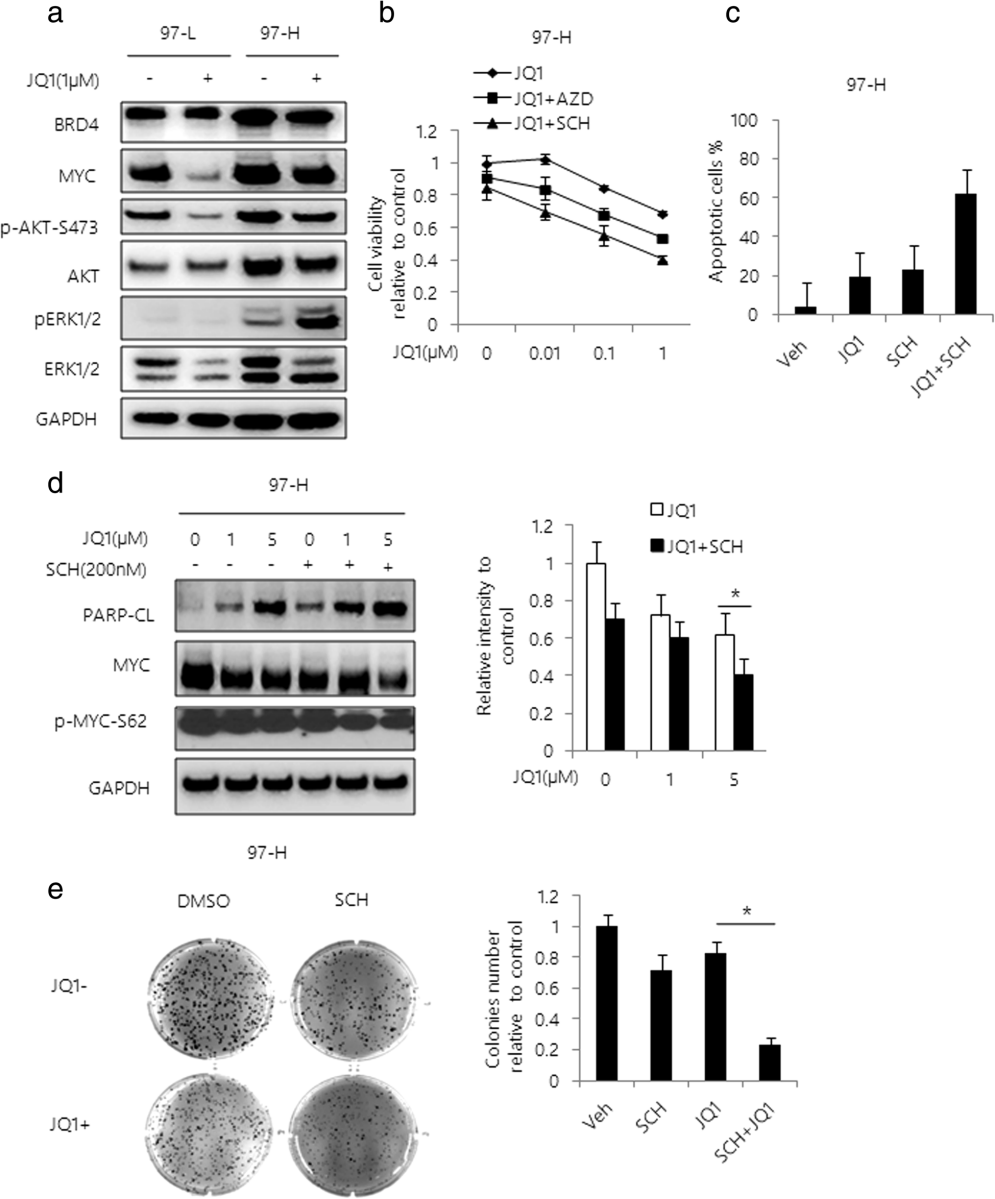
Activation of MAPK pathway mediates JQ1 resistance in HCC. **a** Western blot analysis of 97-L and 97-H cells treated with 1 μM JQ1 for 48 h. Total lysates were subjected to the indicated antibodies. **b** Cell viability curves are shown for varying doses of JQ1 with or without 200 nM SCH or AZD in 97-H cells. Cell viability was determined using Cell Titer-Glo at 48 h after incubation. **c** 97-H cells were treated with vehicle, JQ1, SCH or the combination for 48 h. Apoptosis was assessed and quantified by Annexin V / PI double staining. Quantification of apoptotic cells was determined based on Annexin V positive cells. **d** Western blot analysis of 97-H cells treated with varying doses of JQ1 with or without 200 nM SCH for 48 h. Total lysates were subjectd to the indicated antibodies. Band intensities for MYC were quantified by Image J software and graphed at the right side. **e** Colony formation assays were performed in 6-well plates. 97-H cells were treated with vehicle, 1 μM JQ1, 200 nM SCH or the combination. After 6 weeks of incubation, colonies were stained with crystal violet and the number of colonies per well was determined and graphed at the right side. Data are presented as mean ± s.d. *p < 0.05

As shown above, 97-H cells were relatively resistant to JQ1 compared to 97-L cells. Since 97-L and 97-H cell lines have similar origins, we believed that these two cell lines could be good models to study the mechanism of acquired cellular resistance to JQ1. To investigate the key factors in preventing MYC from proteasomal degradation in JQ1-treated 97-H cells, we evaluated the effects of JQ1 on PI3K/AKT and MAPK/ERK signaling pathways, which have been shown to be associated with JQ1 resistance and MYC posttranscriptional regulation [26]. No significant difference in AKT pathway activity was observed between 97-L and 97-H cells after JQ1 treatment. In contrast, the phosphorylation level of ERK was significantly up-regulated in 97-H cells compared with 97-L cells, with or without JQ1 treatment (Fig. 3a). To determine if inhibition of ERK activity could affect the response of 97-H cells to JQ1, we treated these cells with different concentrations of JQ1 in combination with specific ERK inhibitors, AZD6244 or SCH772984 (SCH) respectively. We found that the combination treatment significantly reduced cell viability and growth in 97-H cells (Fig. 3b). In addition, the Annexin V/PI dual staining also showed that cellular apoptosis was induced by the combined treatment (Fig. 3c and Additional file 5: Figure S5). Furthermore, a remarkable reduction in MYC protein expression and the phosphorylation of MYC protein levels were observed in the combined treatment of JQ1 and SCH (Fig. 3d). The combined treatment also significantly abolished the colony forming ability of 97-H cells, comparing to JQ1 alone (Fig. 3e). Taken together, these results demonstrated that MAPK pathway may be critical for MYC stability and JQ1 resistance.

### Acquired EGFR mutation stabilizes MYC protein in the presence of JQ1

**Fig. 4:**
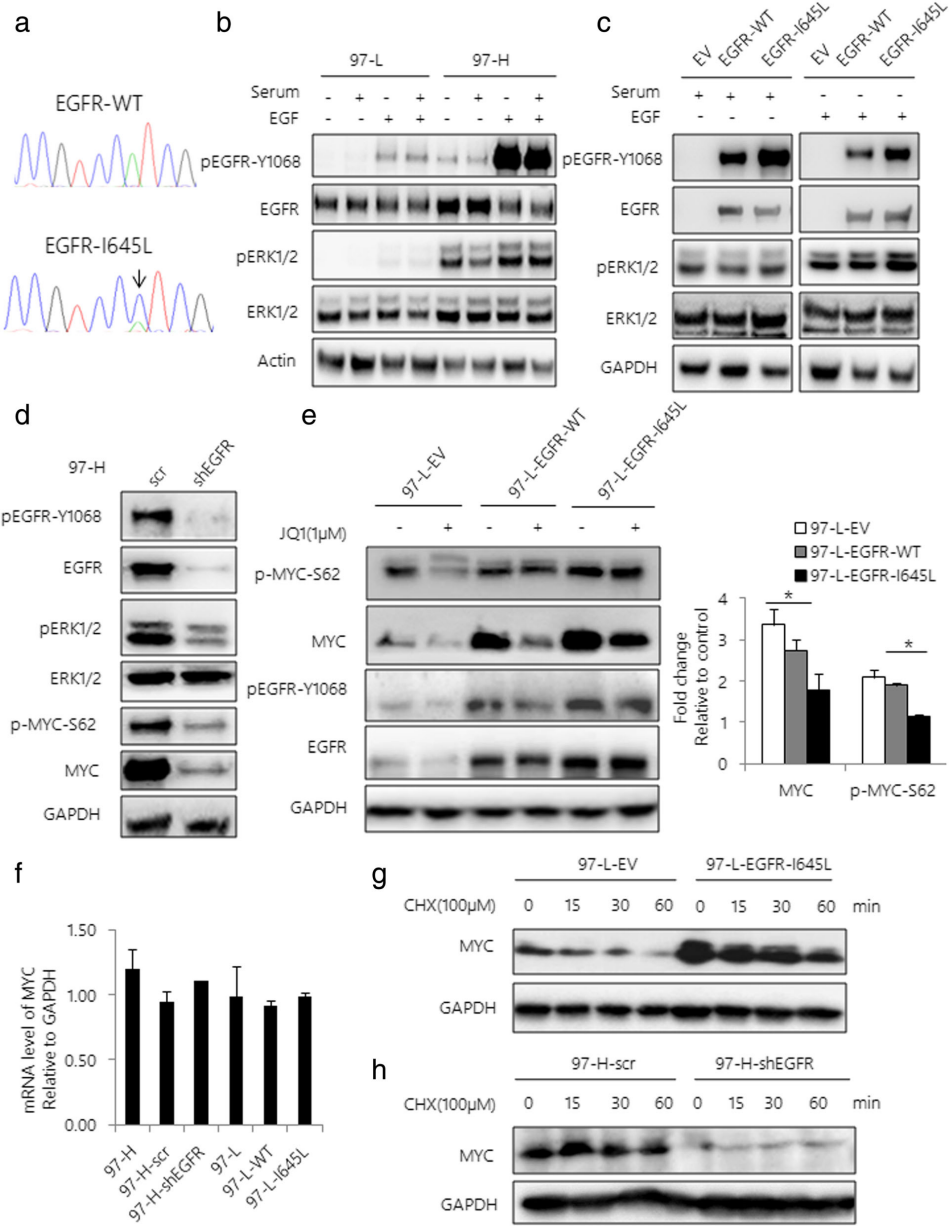
Acquired EGFR mutation confers JQ1 resistance by stabilizing MYC. **a** Diagram of the EGFR-I645L mutation in 97-H cells identified by Sanger sequencing of EGFR mRNA. **b** Western blot analysis of 97-L and 97-H cells with or without EGF stimulation after serum starvation ±10% fetal bovine serum (FBS) for 30 min. Total lysates were subjected to the indicated antibodies. **c** Western blot analysis of EGFR-WT or EGFR-I645L-expressing 293 T cells lysates after serum starvation or EGF stimulation. Total lysates were subjected to the indicated antibodies. EV is the empty vector-transfected 293 T cells. **d** Western blot analysis of 97-H cells transfected with scrambled shRNA or shEGFR. Total lysates were subjected to the indicated antibodies. **e** Western blot analysis of EV, EGFR-WT or EGFR-I645L-expressing 97-L cell lysates after JQ1 treatment. Total lysates were subjected to the indicated antibodies. Band intensities were quantified by Image J software and graphed at the right side. **f** MYC mRNA expression level in 97-H cells transfected with scrambled shRNA or shEGFR and EGFR-WT or EGFR-I645L-expressing 97-L cells were assessed using qRT-PCR. **g** Western blot analysis of 97-L-EV and 97-L-EGFR-I645L cells incubated with 100 μM CHX. **h** Western blot analysis of 97-H-scr and 97-H-shEGFR cells incubated with 100 μM CHX. Data are presented as mean ± s.d. *p < 0.05

To investigate the discrepancy of upstream mechanisms regulating the MAPK pathway in JQ1 insensitive HCC cells, we performed the WES analysis in 97-H and 97-L cells. We identified a previously undescribed EGFR-I645L mutation in 97-H cells. The presence of this mutation was further confirmed by Sanger sequencing (Fig. 4a). Next, to determine if EGFR-I645L is potentially associated with JQ1 resistance in 97-H cell line, we first examined the effects of EGFR-I645L on activation of EGFR and MEK/ERK pathway. As expected, we found that both p-EGFR and p-ERK levels were much higher in 97-H cells than that of 97-L cells, regardless of Extracellular Growth Factor (EGF) status (Fig. 4b). To further characterize the function of this mutation, we over-expressed both EGFR-WT and EGFR-I645L mutant in 293 T cells respectively. In agreement with the results above, expression of EGFR-I645L mutant significantly increased the level of both p-EGFR-Y1068 and p-ERK compared to EGFR-WT under either serum or EGF stimulation, suggesting that EGFR-I645L is an activating mutation (Fig. 4c). To further determine if EGFR-I645L mutant contributes to JQ1 resistance through activating MAPK pathway, we knocked down expression of EGFR in 97-H cells, showing that inhibition of EGFR activity significantly reduced the levels of p-EGFR and p-ERK, subsequently decreased the levels of both MYC and p-MYC-Ser62 (Fig. 4d). In contrast, overexpression of EGFR-I645L mutant increased p-MYC-Ser62 and MYC levels in 97-L cells compared to those expressing EGFR-WT, in the absence or presence of JQ1 (Fig. 4e). Moreover, neither knockdown nor overexpression of EGFR affected MYC mRNA levels (Fig. 4f). Furthermore, expression of EGFR-I645L mutant enhanced the half-life of MYC protein upon the treatment of CHX (Fig. 4g). In contrast, knockdown of EGFR dramatically decreased MYC protein level in the presence of CHX (Fig. 4h). These results suggest that EGFR/ERK activation is critical for stabilizing MYC protein.

### EGFR inhibition sensitizes HCC cells to JQ1

**Fig. 5:**
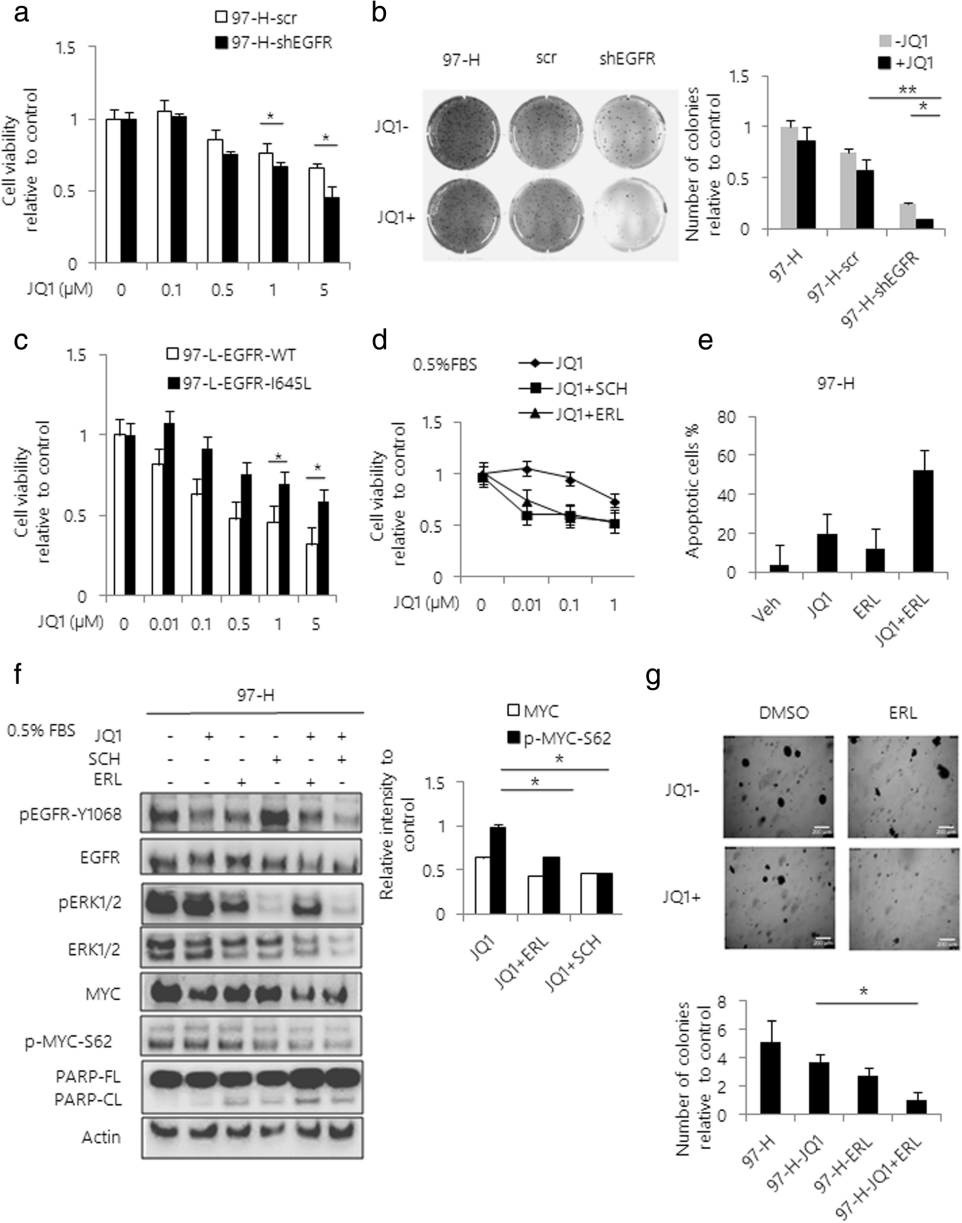
EGFR inhibition sensitizes HCC cells to JQ1. **a** Cell viability curves are shown for varying doses of JQ1 in 97-H cells transfected with scrambled shRNA or shEGFR. Cell viability was determined at 48 h after treatment using Cell Titer-Glo. **b** Colony formation assays were performed in 6-well plates. 97-H cells transfected with scrambled shRNA or shEGFR were treated with 1 μM JQ1 for 6 weeks. After 6 weeks of incubation, colonies were stained with crystal violet and the number of colonies per well was determined and graphed at the right side. **c** Cell viability curves are shown for varying doses of JQ1 in EGFR-WT or EGFR-I645L expressing 97-L cells. Cell viability was determined at 48 h after treatment using Cell Titer-Glo. **d** Cell viability curves are shown for varying doses of JQ1 with or without a fixed dose of SCH or ERL in 97-H cells. **e** 97-H cells were treated with vehicle, JQ1, ERL or the combination for 48 h. Apoptosis was assessed and quantified by Annexin V / PI double staining. Quantification of apoptotic cells was determined based on Annexin V positive cells. **f** Western blot analysis of 97-H cells treated with 1 μM JQ1, 200 nM SCH, 1 μM ERL or the combination of compounds for 48 h. Total lysates were subjected to the indicated antibodies. Band intensities were quantified by Image J software and graphed at the right side. **g** Colony formation assays were performed in 6-well plates. 97-H cells were treated with vehicle, 1 μM JQ1, 1 μM ERL or the combination of two drugs. After 6 weeks of incubation, colonies were stained with crystal violet and the number of colonies per well was determined and graphed at following position Data are presented as mean ± s.d. *p < 0.05, **p < 0.01

Next, to examine if inhibition of EGFR activity could overcome JQ1 resistance in HCC cells, we knocked down EGFR by lentiviral vector-based shRNA infection. We found that loss of EGFR expression significantly enhanced the JQ1-induced growth inhibition in 97-H cells (Fig. 5a). Moreover, MYC protein levels markedly decreased with induction of apoptosis following the treatment with JQ1 in absence of EGFR expression (Additional file 6: Figure S6a). The colony formation assay also confirmed that EGFR inhibition reduced the number of colonies of 97-H cells with JQ1 treatment (Fig. 5b). On the other hand, the expression of EGFR-I645L mutant in 97-L cells attenuated the anti-proliferative effects of JQ1, compared to EGFR-WT (Fig. 5c). To evaluate the synergistic effects, we treated 97-H cells with Erlotinib (ERL), an specific EGFR inhibitor, in combination with varying doses of JQ1. The results showed that the combined treatment of ERL with JQ1 significantly inhibited 97-H cell growth compared to either ERL or JQ1 alone (Fig. 5d). Moreover, the combination of ERL with JQ1 significantly induced the apoptosis of 97-H cells in a dose-dependent manner, which was supported by increased Annexin V + population and PARP cleavage (Fig. 5e, Additional file 6: Figure S6b and 6c). In addition, levels of MYC and p-MYC-Ser62 were effectively inhibited by combination of JQ1 with either ERL or SCH (Fig. 5f). On-target effect of combination treatment was also confirmed by western blot analysis of level of p-ERK and p-EGFR (Fig. 5f). Furthermore, the combined treatment of JQ1 with ERL almost completely abolished the colony forming ability of 97-H cells, while the single drug treatments had limited inhibitory effects (Fig. 5g).

### Combination of JQ1 with EGFR inhibitor effectively reduces JQ1 resistant HCC tumor growth in vivo

**Fig. 6:**
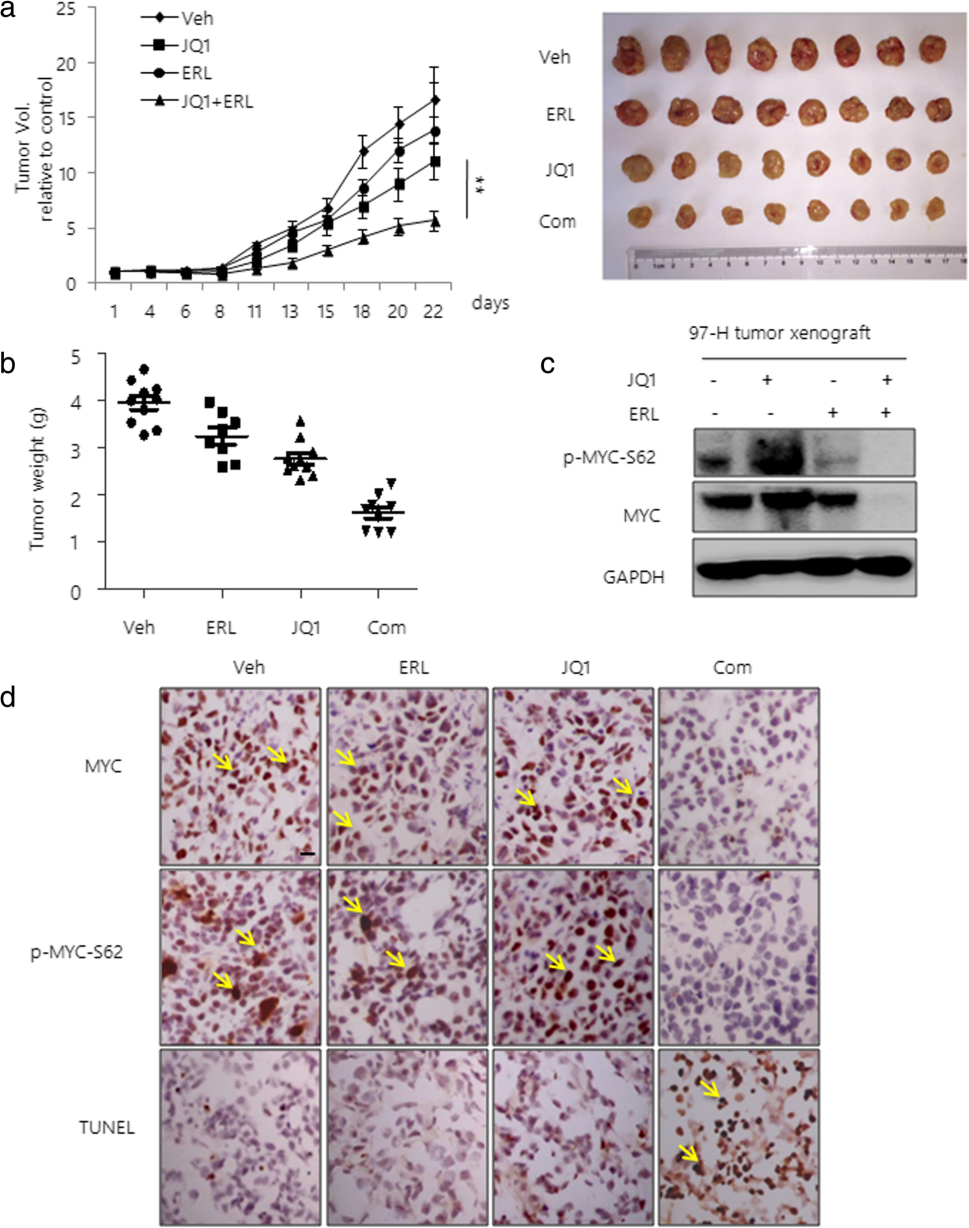
Combination of JQ1 with EGFR inhibitor reduces JQ1 resistant HCC tumor growth. **a** 97-H cells (5 × 10^6^) were injected into the flanks of CB17/SCID mice. After the subcutaneous tumors reached a size of 10 cm^3^, mice were treated with vehicle, JQ1 (50 mg/kg), ERL (50 mg/kg) or a combination of the drugs every 2 days. Tumor volumes were monitored during the treatments. Pictures of tumor removed are shown at the right. **b** Tumor weights are shown after 3 weeks of treatment. **c** Western blot analysis of MYC and p-MYC-Ser62 in tumor lysates. GAPDH was used as a loading control. **d** Representative immunohistochemistry images. Tumors from mice treated with vehicle, JQ1, ERL or the combination were stained with MYC, p-MYC-Ser62 antibodies and TUNEL. Data are presented as mean ± s.d. **p < 0.01, Scale bars, 20 μm

Next, we investigated the efficacy of the combination of JQ1 and EGFR inhibitor in inhibiting HCC tumor growth in vivo. In agreement with our in vitro results, we found that either JQ1 or ERL alone resulted in a modest inhibition of tumor growth. However, a significantly greater anti-tumor effect was observed in the group with combination treatment (Fig. 6a). Average tumor volumes were significantly smaller in mice subjected to the combined treatment compared to either agent alone (Fig. 6b). Consistently, we found that the combined treatment significantly decreased the levels of MYC and p-MYC-Ser62, compared to the JQ1 alone treatment (Fig. 6c and d). Furthermore, induction of apoptosis was also observed in the combined treatment mice group, supported by increased level of TUNEL positive cells (Fig. 6d).

## Discussion

MYC is a master oncogenic driver, regulating transcriptional programs to influence cell proliferation and metabolism in a variety of human cancers [35]. JQ1 is a specific and competitive inhibitor of BRD4, currently being evaluated in phase I and II clinical trials for advanced malignancies [36]. Since sorafenib is the first-line drug approved by FDA, we compared the anti-tumor effect of JQ1 with sorafenib, showing that JQ1 had more promising anti-tumor effects than sorafenib in HCC cells expressing relatively high level of MYC in vitro and in vivo. This result could be possibly interpreted by the high dependency of HCC tumor on glycolysis for energy production and MYC regulation of LDHA and PKM2, which play key roles in driving anaerobic glycolysis [32, 37]. Indeed, we showed that JQ1 more efficiently inhibited mitochondrial glycolysis than sorafenib.

Despite JQ1-induced growth inhibition of MYC-positive HCC cell lines, the responses varied significantly in these cells. Interestingly, we observed that JQ1 more effectively decreased MYC protein levels and induced cellular apoptosis in 97-L cells, comparing to 97-H cells. Since these two cell lines were uniformly originated, we inferred that 97-H cells might be a representative model to investigate the molecular basis of JQ1 resistance in HCC. Notably, our qRT-PCR results showed that JQ1 impaired the BRD4-mediated transcription activation of MYC in both 97-H cells and 97-L cells, suggesting that a mechanism of posttranscriptional modification in stabilizing MYC protein in 97-H cells. Indeed, continious phosphorylation of MYC Ser62 was observed in 97-H cells regardless of JQ1 treatment. This was also supported by the enhanced half-life of MYC in the presence of CHX in these cells, suggesting that sustained MYC activity might be critical for JQ1 sensitivity in 97-H cells. Since MAPK signal is associated with JQ1 responses in tumors, we then examined the MAPK activation in response to JQ1 in 97-H cells [29]. As expected. JQ1 treatment significantly activated p-ERK in 97-H cells. In addition, inhibition of ERK by a specific ERK inhibitor SCH overcame the JQ1 resistance and decreased MYC protein level, suggesting that MAPK pathway might be responsible for stability of MYC protein in HCC cells. This is consistent with previously study that ERK can directly interact with phosphorylated FBW7 to regulate the ubiquitination of FBW7 itself, subsequently preventing MYC degradation [34]. Therefore, it is essential to understand the mechanism of acquired MAPK activation in 97-H cells.

The elevated ERK activity could result from the constitutive activation of upstream molecules, including EGFR and FGFR families, cytokines and hormones [26, 27]. Indeed, our WES results identified a previously undescribed EGFR-I645L mutation in 97-H cell line. Notably, we demonstrated that EGFR-I645L is an activating mutation, supported by the constitutive expression of p-EGFR-Y1068 and p-ERK1/2 regardless of serum or EGF stimulation. More importantly, in combination with the ERK inhibition, blockade of EGFR activity by either RNA interference or EGFR inhibitor effectively decreased the level of p-MYC-Ser62 and reduced cell growth by inducing apoptosis in 97-H cells. In contrast, over-expression of EGFR-I645L mutant remarkably increased the level of p-MYC-Ser62 in 97-L cells compared to the EGFR-WT. Furthermore, combination of JQ1 with EGFR inhibitor significantly reduced 97-H tumor growth in vivo, along with decreased level of p-MYC-Ser62. Taken together, these findings suggest that inhibition of EGFR signal could sensitize HCC cells to JQ1 through attenuating MYC stability in vitro and in vivo.

## Conclusion

In summary, we demonstrated that JQ1 has more potent anti-tumor effects than sorafenib in MYC-overexpressing HCC cells. Furthermore, MAPK activation affects the sensitivity of 97-H cells to JQ1. Mechanistically, we showed that EGFR-I645L, an activating EGFR mutation, impaired the sensitivity of HCC to JQ1 via MAPK regulation of MYC stability. In addtion, combination of JQ1 with EGFR inhibitors significantly enhanced the anti-tumor effect in vitro and in vivo. Since MYC amplification co-occurring with EGFR activation, is frequently observed in advanced HCC (http://www.cbioportal.org), we suggest that EGFR or MAPK status might be considered ahead of JQ1 therapy.

## Additional files

Additional file 1: **Figure S1.** Prognostic analysis of MYC expression in HCC. (a) Analysis of the risk based on MYC expression in HCC using SurvExpress compilation. (b) The overall survival probability based on MYC expression in HCC patients using the Kaplan-Meier analysis. (TIF 239 kb)

Additional file 2: **Figure S2.** BET inhibitor inhibited tumor growth more potently than sorafenib in MYC- positive HCC cells. HCC cells were treated with either JQ1 or sorafenib for 48 h. Apoptosis was assessed by Annexin V / PI double staining. Quantification of apoptotic cells was determined based on Annexin V positive cells. (TIF 300 kb)

Additional file 3: **Figure S3.** JQ1 resulted in a greater reduction of tumor growth than sorafenib in vivo. (a) BEL-7402 and 97-L cells (5 × 10^6^ each) were injected into the flanks of CB17/SCID mice. After the subcutaneous tumors reached a size of 10 cm^3^, mice were randomly treated with vehicle, JQ1 or sorafenib at 50 mg/kg every 2 days. Tumor images is shown. (b) Analysis of apoptosis in 97-L tumor xenografts by IHC staining. Tumors from mice treated with vehicle, JQ1, or sorafenib were stained with H&E, MYC, and TUNEL. Representative immunohistochemistry images were shown. (c) Immunoblot analysis of tumor lysates treated with vehicle, JQ1 or sorafenib, using the indicated antibodies. (TIF 1170 kb)

Additional file 4: **Figure S4.** JQ1 significantly induced apoptosis in MYC-positive HCC cells. HCC cells were treated with JQ1 for 48 h. Apoptosis was assessed by Annexin V / PI double staining. Quantification of apoptotic cells was determined based on Annexin V positive cells. (TIF 413 kb)

Additional file 5: **Figure S5.** Combination of JQ1 with ERK inhibitor induced cellular apoptosis. HCC cells were treated with JQ1, SCH772984 (SCH) or the combination. Apoptosis was assessed by Annexin V / PI double staining. Quantification of apoptotic cells was determined based on Annexin V positive cells. Representative result of FACS analysis was shown. (TIF 196 kb)

Additional file 6: **Figure S6.** Inhibition of EGFR activity overcame the JQ1 resistance. (a) Immunoblot analysis of 97-H cells expressing control shRNA or EGFR shRNA treated with JQ1. (b) HCC cells were treated with JQ1, Erlotinib (ERL) or the combination. Apoptosis was assessed by Annexin V / PI double staining. Quantification of apoptotic cells was determined based on Annexin V positive cells. Representative result of FACS analysis was shown. (c) Immunoblot analysis of 97-H cells treated with variable doses of JQ1 with or without a fixed dose of ERL. Total lysates were subjected to the indicated antibodies. (TIF 514 kb)

## Abbreviations

BET: bromodomain and extra-terminal
BRD4: Bromodomain-containing protein 4
DMSO: Dimethyl sulfoxide
ECAR: Extracellular acidification rate
EGFR: epidermal growth factor receptor
ERK: extracellular regulated protein kinases
HCC: hepatocellular carcinoma
MAPK: mitogen-activated protein kinase
PARP: poly ADP-ribose polymerase
PARP-CL: cleaved parp
PARP-FL: full parp
qRT-PCR: Quantitative real time polymerase chain reaction
scr: scrambled shRNA
Veh: vehicle
WES: Whole Exome Sequencing
WT: wild type
