# Supplementary figures and images for: EGFR signaling confers resistance to BET inhibition in hepatocellular carcinoma through stabilizing oncogenic MYC
Source: J Exp Clin Cancer Res. 2019 Feb 15;38:83. doi: 10.1186/s13046-019-1082-6 (PMC6377788; doi:10.1186/s13046-019-1082-6)

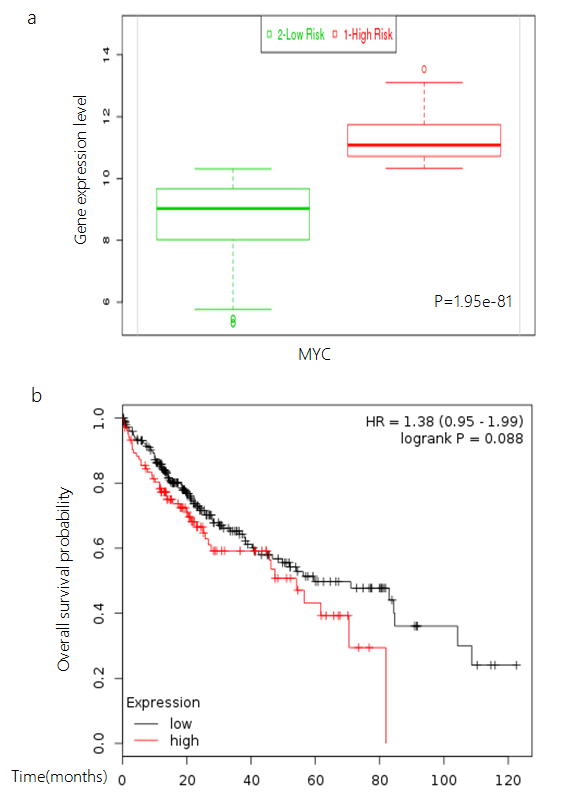

Supplement: Supplementary file 1 — Figure S1. Prognostic analysis of MYC expression in HCC. (a) Analysis of the risk based on MYC expression in HCC using SurvExpress compilation. (b) The overall survival probability based on MYC expression in HCC patients using the Kaplan-Meier analysis. (TIF 239 kb) [file 13046_2019_1082_MOESM1_ESM.tif]

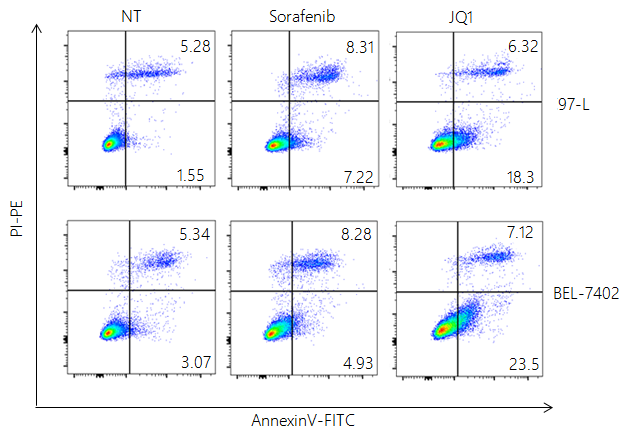

Supplement: Supplementary file 2 — Figure S2. BET inhibitor inhibited tumor growth more potently than sorafenib in MYC- positive HCC cells. HCC cells were treated with either JQ1 or sorafenib for 48 h. Apoptosis was assessed by Annexin V / PI double staining. Quantification of apoptotic cells was determined based on Annexin V positive cells. (TIF 300 kb) [file 13046_2019_1082_MOESM2_ESM.tif]

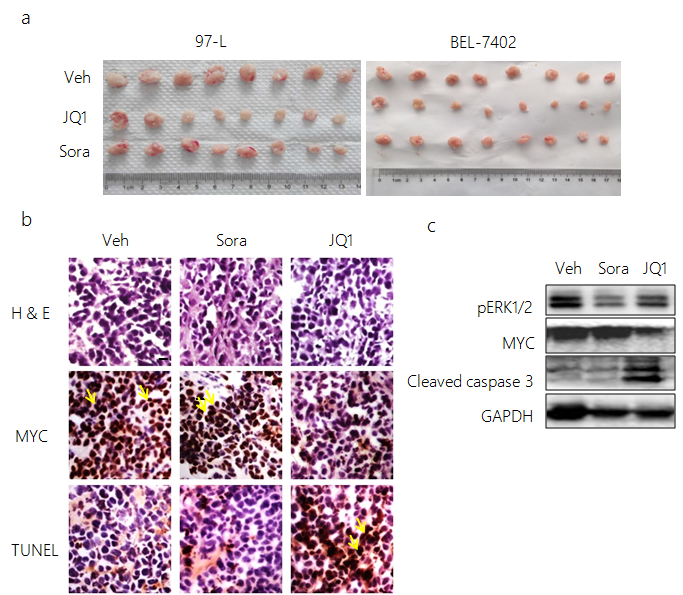

Supplement: Supplementary file 3 — Figure S3. JQ1 resulted in a greater reduction of tumor growth than sorafenib in vivo. (a) BEL-7402 and 97-L cells (5 × 10^6^ each) were injected into the flanks of CB17/SCID mice. After the subcutaneous tumors reached a size of 10 cm3, mice were randomly treated with vehicle, JQ1 or sorafenib at 50 mg/kg every 2 days. Tumor images is shown. (b) Analysis of apoptosis in 97-L tumor xenografts by IHC staining. Tumors from mice treated with vehicle, JQ1, or sorafenib were stained with H&E, MYC, and TUNEL. Representative immunohistochemistry images were shown. (c) Immunoblot analysis of tumor lysates treated with vehicle, JQ1 or sorafenib, using the indicated antibodies. (TIF 1170 kb) [file 13046_2019_1082_MOESM3_ESM.tif]

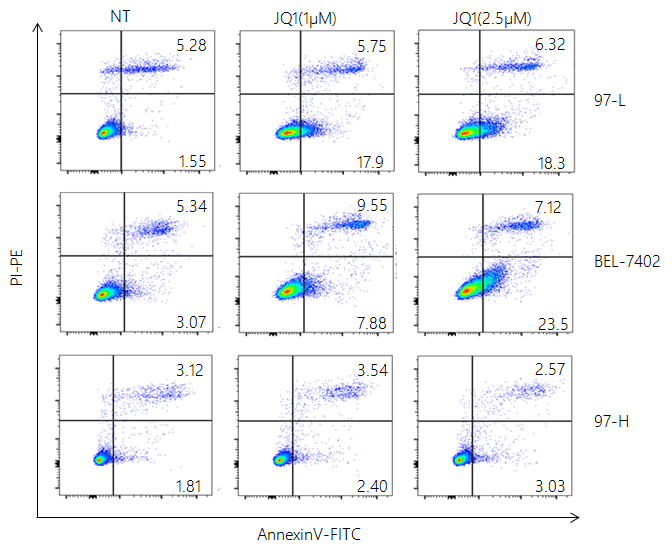

Supplement: Supplementary file 4 — Figure S4. JQ1 significantly induced apoptosis in MYC-positive HCC cells. HCC cells were treated with JQ1 for 48 h. Apoptosis was assessed by Annexin V / PI double staining. Quantification of apoptotic cells was determined based on Annexin V positive cells. (TIF 413 kb) [file 13046_2019_1082_MOESM4_ESM.tif]

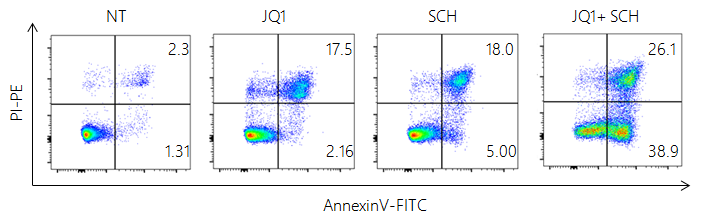

Supplement: Supplementary file 5 — Figure S5. Combination of JQ1 with ERK inhibitor induced cellular apoptosis. HCC cells were treated with JQ1, SCH772984 (SCH) or the combination. Apoptosis was assessed by Annexin V / PI double staining. Quantification of apoptotic cells was determined based on Annexin V positive cells. Representative result of FACS analysis was shown. (TIF 196 kb) [file 13046_2019_1082_MOESM5_ESM.tif]

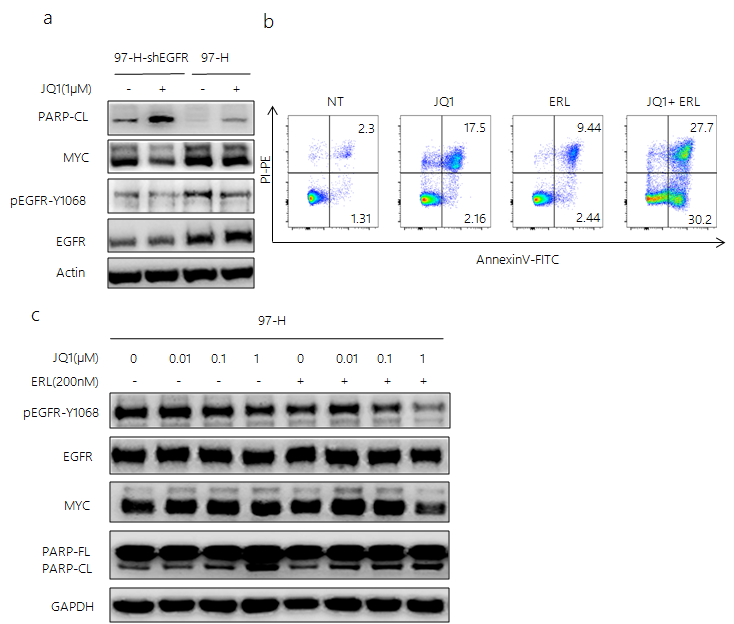

Supplement: Supplementary file 6 — Figure S6. Inhibition of EGFR activity overcame the JQ1 resistance. (a) Immunoblot analysis of 97-H cells expressing control shRNA or EGFR shRNA treated with JQ1. (b) HCC cells were treated with JQ1, Erlotinib (ERL) or the combination. Apoptosis was assessed by Annexin V / PI double staining. Quantification of apoptotic cells was determined based on Annexin V positive cells. Representative result of FACS analysis was shown. (c) Immunoblot analysis of 97-H cells treated with variable doses of JQ1 with or without a fixed dose of ERL. Total lysates were subjected to the indicated antibodies. (TIF 514 kb) [file 13046_2019_1082_MOESM6_ESM.tif]
